# No Difference in Face Scanning Patterns Between Monolingual and Bilingual Infants at 5 Months of Age

**DOI:** 10.1111/desc.70117

**Published:** 2025-12-30

**Authors:** Charlotte Viktorsson, Terje Falck‐Ytter

**Affiliations:** ^1^ Development and Neurodiversity Lab, Department of Psychology Uppsala University Uppsala Sweden; ^2^ Department of Women's and Children's Health, Center of Neurodevelopmental Disorders (KIND), Division of Neuropsychiatry Karolinska Institutet Stockholm Sweden

**Keywords:** bilingualism, eye tracking, infants, language, social attention

## Abstract

It has been suggested that bilinguals take greater advantage of visual speech cues than monolinguals. Therefore, in a sample of 474 (47.3% females) monolingual and 101 (48.5% females) bilingual infants at 5 months of age, we examined the tendency to look at the eyes versus the mouth of dynamic faces, as well as the latency and ratio of looking at a static face interspersed with non‐social objects. No significant differences were found for these measures, suggesting that monolingual and bilingual infants orient to and scan faces in a similar way. Although no association was found between the tendency to look at eyes versus mouth at 5 months and vocabulary at 24 and 36 months, a higher tendency to look at the eyes was related to a larger receptive vocabulary at 14 months, but only in the monolingual group (*β* = 0.15, 95% CI: 0.04; 0.27, *p* = 0.011). However, the difference in beta values of this association between mono‐ and bilinguals was not statistically significant. In conclusion, we did not find support for the hypothesis that bilingual infants rely on visual speech cues from the mouth more than monolinguals do, and there was no association between the tendency to look at eyes versus mouth and later language development in the bilingual group.

**Summary:**

- It has been suggested that bilinguals take greater advantage of visual speech cues than monolinguals.
- Here, no differences between bilingual and monolingual 5‐month‐olds were found regarding any measures of face scanning.
- The findings suggest similar visual attention patterns in mono‐ and bilingual infants, with no impact on bilingual language development.

## 1 Introduction

Faces are important sources of social information, which is reflected in an inherent tendency of human infants to preferentially attend to faces and face‐like stimuli, already at birth (Farroni et al. 2006). When looking at faces, infants mainly attend to the eyes and the mouth (e.g., Viktorsson et al. 2023), but the preference for either of them changes slightly throughout development (see Bastianello et al. 2022 for a review). For example, Lewkowicz and Hansen‐Tift (2012) presented videos of talking faces to 4‐, 6‐, 8‐, 10‐, and 12‐month‐old monolinguals, and found that the infants attended more to the eyes at 4 months, equally to the eyes and mouth at 6 months, and more to the mouth at 8 and 10 months. At 12 months, they attended more to the mouth when hearing a non‐native language than when hearing their native language. The timepoint of the shift in attention from the eyes to the mouth coincides with the emergence of canonical babbling (Long et al. 2024), suggesting that this shift in attention potentially reflects a mechanism by which the infant uses audiovisual speech cues to facilitate language acquisition. Lewkowicz and Hansen‐Tift (2012) hypothesized that a stronger tendency to look at the mouth at 12 months in response to hearing a non‐native language reflected the need to rely on salient visual speech cues to help them discern the unfamiliar speech. In line with this, a higher tendency to look at the mouth during the second half of the first year has been associated with concurrent expressive language ability (Tsang et al. 2018) and larger expressive vocabulary during toddlerhood (Imafuku and Myowa 2016; Tenenbaum et al. 2015; Young et al. 2009). In contrast, during the first months of life, when there is generally a stronger tendency for infants to attend to the eyes rather than the mouth, a higher ratio of looking to the eyes and less looking to the mouth has been associated with higher receptive vocabulary in toddlerhood (Viktorsson et al. 2023; Cruz et al. 2020).

Infants who grow up bilingual are able to successfully integrate and develop two different languages. In order to succeed, it has been hypothesized that bilingual infants take greater advantage of the redundancy of the audiovisual speech that they usually experience during social interactions. Since bilingual infants must clearly identify both of their native languages while also keeping them distinct, they may rely on audiovisual speech cues more than monolingual infants. In particular, bilingual infants might focus on a speaker's mouth at an earlier age and more often during the initial stages of learning two languages, helping them understand the unique characteristics of each. In a study by Pons et al. (2015), 4‐, 8‐, and 12‐month‐old monolingual and bilingual infants were presented with videos of talking faces that spoke either the infants’ native language or a language that was non‐native to them. Monolingual infants exhibited the same visual pattern as in Lewkowicz and Hansen‐Tift (2012), but the bilingual infants shifted their attention to the mouth earlier in development that the monolingual infants, and continued to preferentially attend to the mouth even at 12 months of age. They also attended more to the mouth in response to a non‐native language than the monolingual infants did. As additional support for the notion that speech cues provided by the mouth of a talking face provides important information for differentiating languages, it has been found that 8‐month‐old bilinguals, but not monolinguals, can differentiate between two different languages when viewing silent videos of talking faces (Weikum et al. 2007), even when both languages are non‐native to the infants (Sebastian‐Galles et al. 2012). It has also been found that 15‐month‐old bilinguals who are exposed to two phonetically similar languages in their daily life attend more to the mouth than bilingual toddlers who are exposed to two distant languages in their daily life (Birules et al. 2019), suggesting that reliance on visual speech cues partly depends on the difficulty of the speech‐processing task. In addition, 8‐month‐old bilinguals also attend more to the mouth when seeing videos of different emotional expressions than monolinguals do (Ayneto and Sebastian‐Galles 2017), and 15‐month‐old monolinguals learn to anticipate a movement at the eye region of a dynamic face, while bilinguals fail to disengage from the mouth (Fort et al. 2018). However, some studies have not found any difference in attention to eyes and mouth in bilingual versus monolingual children at 6–12 months of age (Tsang et al. 2018) or at 5 months to 5 years of age (Morin‐Lessard et al. 2019). It is notable, though, that several studies on this topic have included small sample sizes (e.g., five monolingual infants vs. eight bilingual infants; Schonberg et al. 2014), and few studies have analyzed the gaze of infants in the first months of life, or examined potential differences between mono‐ and bilinguals in the association between early attention to eyes and mouth, and later language development.

When presenting static images of faces interspersed with non‐social objects, 5–6‐month‐olds preferentially orient toward the face (Gliga et al. 2009; Portugal et al. 2024), and maintain their attention to the face longer than to non‐social objects (Di Giorgio et al. 2012). In a recent study, Mercure et al. (2018) found that 4–10‐month‐old bilingual infants showed a significantly shorter latency of first look to faces, as compared to monolingual infants, when being shown static images of faces interspersed with non‐social objects. Bilingual infants also had a significantly higher number of fixations to the face, and they, descriptively, had higher total fixation duration to faces, but this difference was not statistically significant. In a follow‐up study of 15‐ to 18‐month‐olds, bilinguals oriented significantly faster to faces than monolinguals, but there was no difference in number of fixations on faces (Mousley et al. 2023).

Here, in a sample of 575 infants, we aimed to analyze different aspects of face processing in 5‐month‐old monolinguals and bilinguals. We hypothesized that bilinguals, at 5 months, would have a significantly lower eye‐mouth‐index (EMI) when viewing dynamic videos of faces than monolinguals, meaning that they tend to look more at the mouth relative to eyes (*H1*). In an earlier analysis of the same sample as in the current study, we found a weak but significant positive association between a tendency to look at the eyes at 5 months and receptive vocabulary at 14 months (Viktorsson et al. 2023), when analyzing the total sample without taking language exposure into account. However, as it has been hypothesized that bilingual infants look more at the mouth than monolinguals in order to facilitate language acquisition, and a stronger preference for the mouth has been found already at 4.5 months of age (Pons et al. 2015), an association between mouth‐looking and later language development could be expected in bilinguals already at 5 months of age. Therefore, we hypothesized that we would find significantly higher (in the positive direction) beta estimates for monolinguals than bilinguals regarding the association between EMI at 5 months and receptive vocabulary at 14 months, as well as expressive vocabulary at 24 and 36 months (*H2*).

In an earlier study, we found that the tendency to look at eyes versus mouth of talking faces and the ratio of looking at a static face interspersed with non‐social objects are only moderately correlated (Portugal et al. 2024), meaning that these measures do not reflect the same aspects of social attention. Although there is no clear theoretical rationale as to why bilinguals would orient faster to faces or maintain their attention to faces longer than monolinguals, we aimed to, as an additional analysis, examine whether we would find the same patterns as in Mercure et al. (2018). Therefore, we hypothesized that bilingual infants, at 5 months, would have a significantly shorter latency of first look to a static face interspersed with non‐social objects, as compared to monolingual infants (*H3*). In addition, we hypothesized that bilingual infants, at 5 months, would have a significantly higher face preference (when interspersed with non‐social objects) than monolingual infants (*H4*). All hypotheses and analyses were preregistered at OSF (https://osf.io/bnyqe/).

## 2 Methods

### 2.1 Participants

The sample in this study is a part of the Babytwins Study Sweden (BATSS), which in total consisted of 622 same‐sex twins (311 pairs) that were recruited from the greater Stockholm area (an urban/suburban environment) via the national population registry. Of the invited families, 29% participated in BATSS. Collection of eye tracking data was performed the Centre of Neurodevelopmental Disorders at Karolinska Institutet in Stockholm, at the initial 5‐month lab visit. Follow‐up questionnaires were administered online at 14, 24, and 36 months. Parents gave informed consent to take part. BATSS was approved by the regional ethics board in Stockholm and was conducted in accordance with the Declaration of Helsinki.

General exclusion criteria for BATSS were opposite‐sex twin pairs, known presence of genetic syndrome related to ASD, uncorrected vision or hearing impairment, very premature birth (prior to week 34), diagnosis of epilepsy, presence of developmental or medical condition likely to affect brain development (e.g., Cerebral Palsy), and infants where none of the biological parents were involved in the infant's care. Among the recruited and tested infants, three twins were excluded from analysis because they subsequently were found not to fulfil the general criteria (above) due to seizures at the time of birth and spina bifida. In addition, for this analysis we excluded infants due to twin‐to‐twin transfusion syndrome, birthweight below 1.5 kg, and twins with no caregiver fluent in Swedish (*n* = 13 pairs and 1 twin). One twin pair was additionally excluded due to not having valid data on language status. After exclusions based on eye tracking data quality (see below), the final sample consisted of 575 infants, who contributed to at least one analysis. The exact number of infants included in each eye tracking condition is reported in the section *Eye tracking measures*. There were no statistically significant differences between included and excluded infants regarding sex (*p* = 0.062), age (*p* = 0.944), education level of the caregivers (*p* = 0.239), or family income (*p* = 0.250).

Based on a recent review of bilingualism in infancy and toddlerhood (Rocha‐Hidalgo and Barr 2023), the most common used cutoffs for the definition of bilingualism is that the child is exposed to a second language for at least 20%–25% of the time. Therefore, our definition of bilingualism is that the child hears a second language at home for at least 20% of the time, as reported by their caregiver. This single‐question approach to measure bilingualism in young children has been used in previous studies (e.g., Xuan and Dollaghan 2013), and although parental questionnaires may appear as rather subjective, a recent study compared parent‐rated questionnaires to three full‐day recordings on language input of 10‐month‐old infants and found a high correlation between the two measurements (*r* = 0.77, Orena et al. 2020).

In our study, bilingualism was assessed during an interview at the 5‐month lab visit. The caregiver was asked to specify what language(s) the child hears at home, and the proportion of time at home the child hears each language (0%–100%). The interview did not specify whether the heard speech was directed at the infant or not, the question was simply whether the child hears the language. Using the 20% cutoff, 474 children in our sample were defined as monolingual and had eye tracking data from at least one of the tasks. In total, 101 infants heard a second language at home for at least 20% of the time and had eye tracking data from at least one of the tasks (see below for detailed inclusion criteria of eye tracking data). Of the 101 bilingual infants, 23 also heard a third language at home. These were included in the bilingual group, but sensitivity analyses were performed to examine whether they differed from the bilingual group regarding the primary eye tracking measures (see Results section). In the monolingual group (*n* = 474), 443 infants heard Swedish as the main language at home. The main languages of the rest of the infants in the monolingual group were English, Albanian, Assyrian, Bosnian, Finnish, German, Hindi, Hungarian, Norwegian, Persian, Polish, Russian, and Urdu. Specific number of participants hearing each language is omitted as not to be identifiable. In the bilingual group (*n* = 101), 69 infants heard Swedish as the main language at home. The main languages of the rest of the bilingual group were English, Amharic, Arabic, Chinese, Danish, Greek, Icelandic, Persian, Romanian, Spanish, Tigrinya, Turkish, and Uighur. The main second languages heard in the bilingual group were Swedish (*n* = 26), English (*n* = 14), Amharic, Croatian, Danish, Dutch, Estonian, Farsi, Finnish, French, German, Italian, Cantonese, Kurdish, Lithuanian, Norwegian, Persian, Portuguese, Punjabi, Romanian, Russian, Spanish, Swahili, Syrian, Tagalog, Turkish, Turkmenian, and Urdu. Specific number of participants hearing each language is omitted as not to be identifiable. All infants included in the study had at least one parent who reported that they speak Swedish fluently (although they might not speak Swedish to their children).

The sample is quite diverse in terms of language exposure, which reflects the fact that all infants live in a linguistically diverse area. We included all infants regardless of which language they were exposed to, as we believe that it contributes to ecological validity of the study, and because it is an approach that is in line with earlier studies of bilingual children from linguistically diverse cities (e.g., Poulin‐Dubois et al. 2011).

Although ethnicity of the children was not collected, country of birth was collected from both parents. Based on this information, 100 infants (17.4%) had one parent born in Sweden, and 417 infants (72.5%) had two parents born in Sweden. In total, 58 children (10.1%) had no parent born in Sweden. Information on specific parental birth countries and combination of countries for these children is omitted as not to be identifiable.

Descriptive statistics of the sample is shown in **Table** **1**.

**TABLE 1:** Descriptive statistics of demographic variables.

	Mean (SD)^a^[Min; Max]		
	Monolinguals (*n* = 474)	Bilinguals (*n* = 101)	Group comparisons^b^ *p* value (Cohen's *d*)
***N* females (%)**	224 (47.3%)	49 (48.5%)	0.819 (0.025)
**Age** ^c^ **(in days) at 5‐month assessment**	167.1 (8.5) [145; 203]	169.8 (9.2) [152; 187]	0.004^*^ (−0.321)
**Gestational age**	36.76 (1.18) [34; 39]	36.67 (1.13) [34; 38]	0.502 (0.074)
**Family income** ^d^	6.62 (2.27) [1; 10]	6.55 (2.60) [2; 10]	0.794 (0.032)
**Education level** ^e^	3.30 (0.73) [1.5; 4]	3.33 (0.74) [1.5; 4]	0.716 (−0.040)

^a^ Except for *N* females, which shows the frequency

^b^ Independent‐samples *t*‐test.

^c^ For 11 infants, the age differed between the EMI task and the face pop‐out task, because they did the eye tracking tasks at different days. In these cases, the mean age was used.

^d^ Family income per month. Scale 1–10 where 1 = <20K, 2 = 20–30K, 3 = 30–40K, 4 = 40–50K, 5 = 50–60K, 6 = 60–70K, 7 = 70–80K, 8 = 80–90K, 9 = 90–100K, and 10 = >100K (SEK).

^e^ Mean education level (of both parents) on a scale from 1 to 4, where 1 = Primary, 2 = Secondary, 3 = Undergraduate (≤3 years), and 4 = Postgraduate level (>3 years).

* *p < *0.05

### 2.2 Eye Tracking Measures

#### 2.2.1 Eye‐Mouth‐Index

Gaze data for the eye‐mouth index (EMI) was collected using a Tobii T120 Eye‐tracker, operating at a sampling rate of 60 Hz. Infants were seated approximately 60 cm from the screen, either in a baby chair or on a parent's lap. Before the eye‐tracking session, a 5‐point calibration video was shown, and the experiment only began after successful calibration. At the start of the session, an additional 5‐point video was displayed once to validate the calibration offline.

The stimuli consisted of 20 videos of a women either singing, talking (common nursery rhymes), or just smiling to the camera (two women contributed equally to all conditions). In the videos where they were singing or talking, the language used was Swedish. The length of the videos ranged from 4 to 12 s. We found very high correlations of the EMI across conditions (singing, talking, still), ranging from 0.87 to 0.93. Correlations were of similar magnitude when comparing monolinguals (range 0.87–0.92) and bilinguals (range 0.89–0.94). Therefore, we created a mean EMI variable consisting of data from all valid trials, regardless of condition, and used this variable in all further analyses.

**FIGURE 1:**
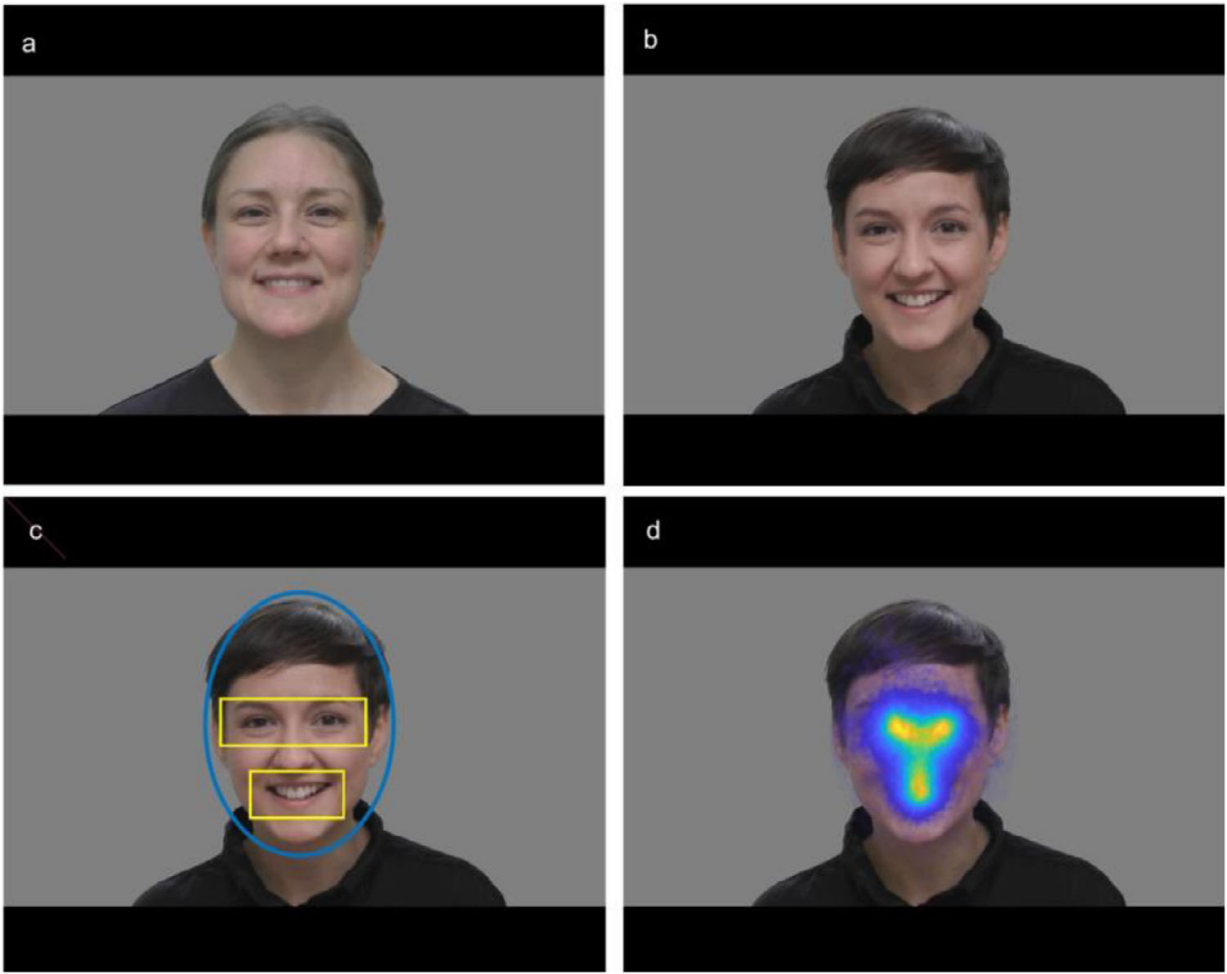
Experimental stimuli. a,b) The videos consisted of a set of face stimuli with the natural voice sound included, from two different models. c) Areas of Interests (AOIs) used for analysis. d) An aggregated gaze heatmap of data from all infants from all stimuli superimposed on one stimulus. The figure is reprinted with permission from Viktorsson et al. (2023).

Preprocessing of gaze data was done using custom scripts written in MATLAB. Fixation filters were not applied, as the EMI is based on accumulated looking time. Data from the additional 5‐point calibration was evaluated via visual inspection after data collection was finished, and a linear transformation of data was applied whenever linear drifts were detected. Based on the visual inspection, the calibration from each infant was classified as either good, good after correction, or unclear. Dynamic areas of interest (AOIs, i.e., face, eyes, and mouth) were created to move in coordination with the stimuli (using custom scripts in MATLAB), and were validated through visual inspection of their coordinates superimposed on the video stimuli. The face AOI was an ellipse with a horizontal radius of 200 pixels and a vertical radius of 280 pixels. Both the mouth AOI and eyes AOI were rectangles, 200 × 100 pixels and 310 × 100 pixels, respectively (see **Figure** **1**).

The EMI had a scale of 0‐1, and was calculated as the mean amount of gaze in the eyes AOI divided by the mean amount of gaze to both the eyes AOI and the mouth AOI (i.e., 1 = looking at the eyes 100% of the time; 0 = looking at the mouth 100% of the time). This measure has been used in earlier studies (Young et al. 2009), and it is independent of differences in total gaze or stimuli duration. An aggregated heatmap of gaze data inside the face AOI (from all infants for all trials) verified that the infants focused mainly on the mouth and the eyes region (Figure 1d).

We then used the results from infants with good calibration data (including after linear transformation) and implemented steps to exclude trials based on general distribution properties. Values were obtained for the 10th percentile for time spent looking at the screen, the 10th percentile for the ratio of looking at the face (relative to the screen) and the 15th percentile for the ratio of looking at the eyes and mouth combined (relative to the face) for each trial. If an infant was below one of these cutoffs on a particular trial or if they looked at the screen for less than 1000 ms (in total), the trial was deemed invalid (regardless of the classification of the calibration). These cutoffs were chosen to minimize data loss while ensuring sufficient data quality within each trial. Infants were included in further analyses if they have at least four valid trials (from any condition). In total, 38 twins were excluded due to not having enough valid data. In addition, some infants did not provide any data due to lack of time, technical reasons, lack of room, or infant being too tired or too fussy (in total, seven pairs and five twins). The final sample for the EMI task consisted of 533 infants.

#### 2.2.2 Face Pop‐Out Task

**FIGURE 2:**
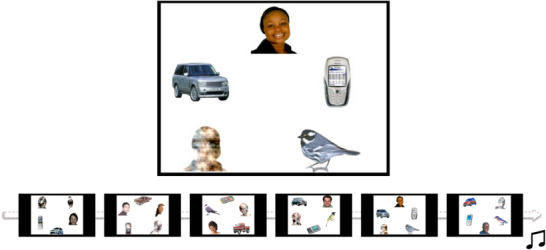
**Face pop‐out condition**. Each image consisted of five pictures, of which one was a face and one was a noise stimulus made from the face. The images were presented in a fixed order shown in the scheme. The figure is reprinted with permission from Portugal et al. (2024).

Gaze data for the face pop‐out task was recorded using a Tobii TX300 Eye tracker, operating at a sampling rate of 120 Hz. A 5‐point calibration video was presented before the eye tracking session, and the experimental task did not begin until a successful calibration was achieved. The stimuli consisted of six different displays of objects, including one face (three males and three females, with the location being counterbalanced within the array; see **Figure** **2**). The non‐social objects consisted of a mobile phone, a bird, a car, and a noise stimulus created from the same face as shown in that array. Each image was shown for 20 s. Before each trial, a central animation was presented, and the stimulus picture was not shown until the infant looked at the central animation, to make sure that their gaze was centered on the screen at the start of the trial.

**FIGURE 3:**
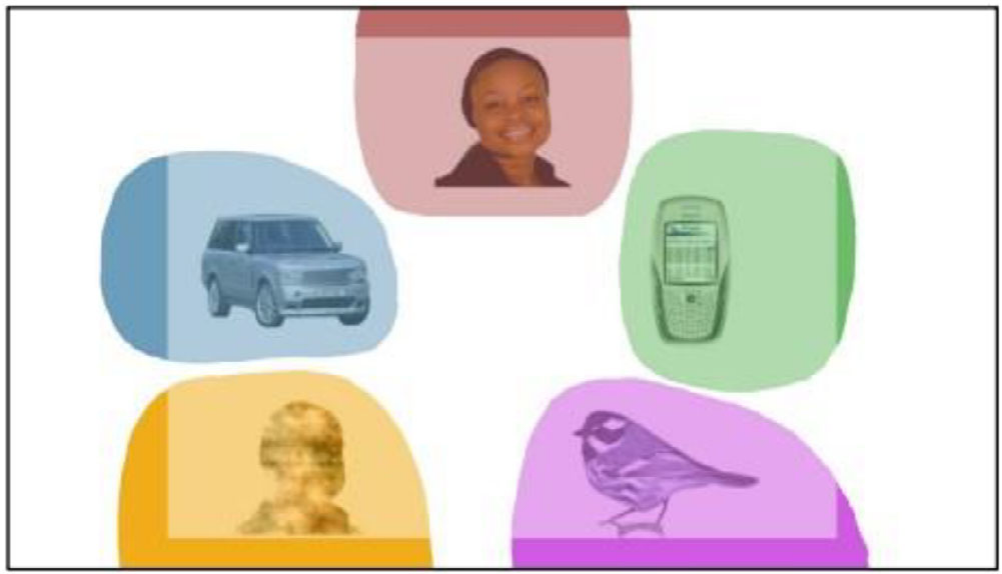
A stimuli image with the AOIs plotted over the face and non‐social objects. The figure is reprinted with permission from Portugal et al. (2024).

Gaze data for the face pop‐out tasks were processed using custom‐written MATLAB scripts (see **Figure** **3** for an example of a stimuli image with overlaying AOIs). Full details on preprocessing steps are reported in Portugal et al. (2024). Trials were excluded if the proportion of valid data was less than 25%, if the total duration of looking time at the screen was less than 5 s, or if the infant did not look at any of the AOIs. The final sample for the face pop‐out task was 532 infants.

The measures used in this study were mean latency (across valid trials) of first look toward a face, and face preference (calculated as total looking time at the face AOI divided by the sum of the looking time at all AOIs averaged across valid trials).

Exclusion criteria for the EMI and the face pop‐out task followed the criteria established in previous studies using the same paradigms (Viktorsson et al. 2023; Portugal et al. 2024), with slight differences in thresholds reflecting task‐specific features such as trial length and number of stimuli.

### 2.3 Parent‐Rated Questionnaires

The MacArthur Communicative Development Inventory, Swedish edition (SE‐CDI; Fenson et al. 1993; Berglund and Eriksson 2000) is a parent‐rated questionnaire that assesses early language development. The Words and Gestures form was administered at 14 months, and the Words and Sentences form was administered at 24 and 36 months. As a measure of receptive vocabulary at 14 months, we used the total Comprehension Score, which is the number of words (out of 382 words) that the infant can understand (but not necessarily produce). At 24 and 36 months, we used the vocabulary checklist score as a measure of expressive vocabulary. The Words and Gestures form included the following statement in the beginning: “If the child's primary language at home is something other than Swedish, you should fill in the questionnaire based on the child's level in the language you speak at home. We are interested in the child's language development regardless of which language the child is learning at home.”, prompting the parents to fill in the questionnaires based on any languages the child knows. Due to being too old at the time of assessment, one twin pair was excluded from the 14‐month timepoint (aged 516 days), and one pair was excluded from the 24‐month timepoint (aged 919 and 920 days, respectively).

### 2.4 Statistical Analyses

The primary analyses of differences in gaze behavior between monolingual and bilingual infants were performed using mixed‐effects models clustered on family ID (which is unique for each twin pair), to account for the fact that twins are not independent of each other. Language status (i.e., monolingualism or bilingualism) was entered as a fixed factor with two categories. Gaze measures (i.e., face preference, latency of first look to faces, and EMI) were entered as outcome variables in three separate models. Age and sex were included as covariates. We also performed sensitivity analyses where we re‐did the main statistical analyses including only infants with Swedish as the main language heard at home.

Prior to analyses, residual plots for the gaze measures were inspected, indicating some deviations from normality. However, linear mixed‐effects models are generally robust to such violations, particularly with larger samples, and no clear patterns suggesting model misspecification were observed.

Associations between the EMI at 5 months and follow‐up measures at 14, 24, and 36 months were calculated separately for mono‐ and bilinguals using the robust sandwich estimator in generalized estimating equations (GEE) in order to account for the correlation between twins in a pair (Carlin et al. 2005). Age and sex were regressed out before the analyses. We then calculated the difference between the beta estimates for monolinguals and bilinguals, the standard error of the difference, and performed a *Z*‐test to determine if the difference was statistically significant.

## 3 Results

No statistically significant differences were found between the bilingual and multilingual infants regarding the EMI (*p* = 0.211), ratio of looking at the face in the face pop‐out task (*p* = 0.253), or latency of first look to faces (*p* = 0.161). Therefore, the multilingual infants were included in the group of bilingual infants for all further analyses. Descriptive statistics of eye tracking measures and follow‐up language measures are shown in **Table** **2** (see **Figures** S1–S3 for distributional plots).

**TABLE 2:** Descriptive statistics of eye tracking measures and language measures.

	Mean (SD) [Min; Max]			
	Monolinguals	*N* monolinguals	Bilinguals	*N* bilinguals
**EMI task (5m)**				
Ratio of looking at face	0.956 (0.024) [0.868; 0.995]		0.956 (0.021) [0.885; 0.990]	
Eye‐mouth‐index	0.704 (0.313) [0.002; 1.000]	437	0.750 (0.295) [0.013; 0.999]	96
Valid trials (*n*)	15.24 (4.41) [4; 20]		15.40 (4.34) [5; 20]	
**Face pop‐out task (5 m)**				
Latency of look to face	2.96 (1.83) [0.41; 10.80]		2.89 (1.79) [0.64; 10.21]	
Ratio of looking at face	0.435 (0.138) [0.090; 0.807]	436	0.438 (0.158) [0.147; 0.790]	96
Valid trials (*n*)	5.78 (0.51) [4; 7]		5.75 (0.54) [4; 6]	
**Language measures** ^a^				
Receptive voc. (14 m)	79.48 (58.58) [1; 308]	344	98.55 (89.02) [5; 375]	66
Expressive voc. (24 m)	211.88 (156.97) [9; 689]	277	177.04 (144.87) [1; 560]	56
Expressive voc. (36 m)	540.82 (151.67) [40; 710]	298	514.48 (132.73) [191; 681]	58

^a^ These measures only include individuals with EMI data, as the language measures are analyzed in relation to that eye tracking data.

### 3.1 The EMI Task

Although the bilingual infants had a descriptively higher EMI (mean = 0.75) than the monolingual infants (mean = 0.70), there was no statistically significant difference between the two groups (**Table** **3**). Likewise, this difference was not statistically significant when including only infants with Swedish as the main language heard at home (B = −0.059, SE = 0.048, t(238.58) = −1.226, *p* = 0.221, see **Table** **S1** for full results).

**TABLE 3:** Results of the linear mixed effects model predicting EMI.

Predictor	Estimate	SE	df	*t*	*p* value	95% CI
Intercept	0.419	0.320	288.467	1.310	0.191	−0.211; 1.049
Language status	−0.052	0.042	290.039	−1.235	0.218	−0.134; 0.031
Sex	0.034	0.032	289.288	1.072	0.285	−0.029; 0.097
Age	0.002	0.002	287.875	0.915	0.361	−0.002; 0.005

*Note*: Model fit: −2LL = 230.65.

### 3.2 The Face Pop‐Out Task

The mean ratio of looking at the face in the face pop‐out task were similar in the monolingual and bilingual group (mean = 0.435 and 0.438, respectively) and the linear mixed effects model showed that there was no statistically significant difference between the two groups (**Table** **4**). Similarly, no significant group difference was found when including only infants with Swedish as their primary language (B = 0.005, SE = 0.022, t(241.65) = 0.211, *p* = 0.833, see **Table** **S2** for full results).

**TABLE 4:** Results of the linear mixed effects model predicting ratio of looking at the face in the face pop‐out task.

Predictor	Estimate	SE	df	*t*	*p* value	95% CI
Intercept	0.177	0.142	285.340	1.251	0.191	−0.101; 0.456
Language status	0.003	0.019	283.690	0.139	0.890	−0.034; 0.039
Sex	−0.023	0.014	284.466	−1.597	0.111	−0.051; 0.005
Age	0.002	0.001	286.548	2.123	0.035	0.000; 0.003

*Note*: Model fit: −2LL = −588.39.

The mean latency of looking at the face in the face pop‐out task were similar in the monolingual and bilingual group (mean = 2.96 and 2.89, respectively) and, again, the linear mixed effects model showed no statistically significant difference between the two groups (**Table** **5**). Similarly, no significant group difference was found when including only infants with Swedish as their primary language (B = 0.054, SE = 0.264, t(224.16) = 0.206, *p* = 0.837, see **Table** **S3** for full results).

**TABLE 5:** Results of the linear mixed effects model predicting latency of looking at the face in the face pop‐out task.

Predictor	Estimate	SE	df	*t*	*p* value	95% CI
Intercept	7.432	1.688	255.210	4.403	<0.001	4.108; 10.756
Language status	0.012	0.222	255.227	0.056	0.956	−0.424; 0.449
Sex	0.098	0.170	256.158	0.577	0.565	−0.237; 0.433
Age	−0.028	0.010	256.504	−2.840	0.005	−0.047; −0.008

*Note*: Model fit: −2LL = 2143.15.

### 3.3 Associations With Language Measures

**FIGURE 4:**
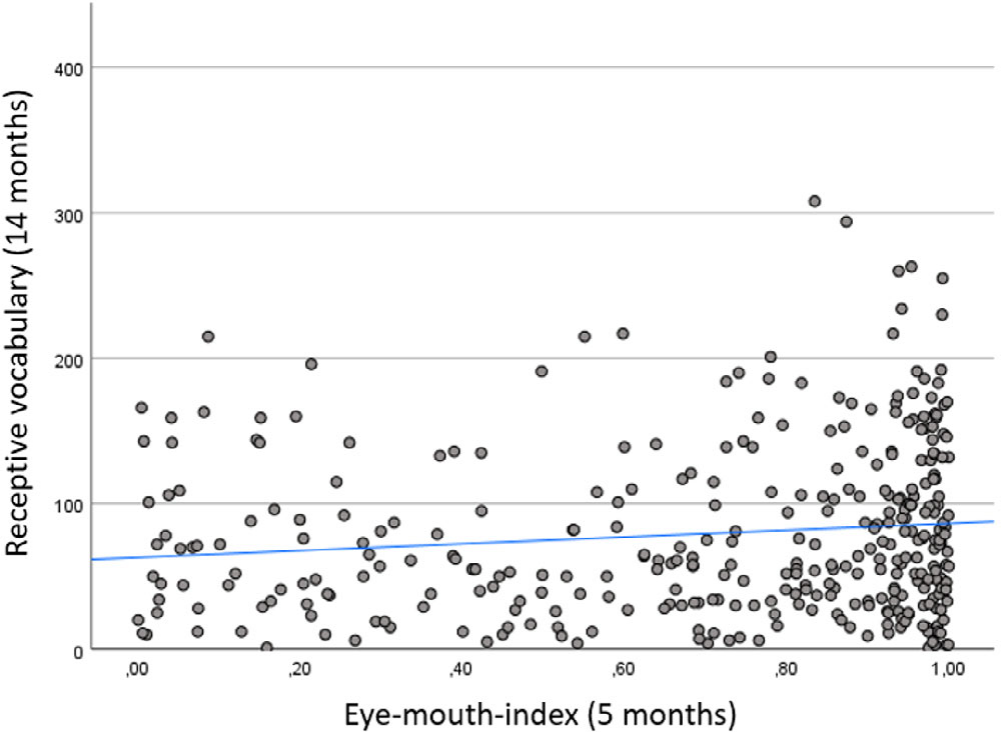
A scatterplot of the association between the EMI at 5 months and receptive vocabulary at 14 months in the monolingual group. Plotted with a regression line for illustrative purposes, but note that the statistical analysis accounted for similarities between twins (not shown in figure).

First, we analyzed potential group differences in the CDI measures at 14, 24, and 36 months. There were no differences between monolinguals and bilinguals with regards to receptive vocabulary at 14 months (t(76.152) = −1.672, *p* = 0.099), expressive vocabulary at 24 months (t(331) = 1.534, *p* = 0.126), or expressive vocabulary at 36 months (t(354) = 1.233, *p* = 0.218). There was only one statistically significant association between the EMI and later language measures (**Table** **6**). This association was positive, and only found in the monolingual group, between EMI at 5 months and receptive vocabulary at 14 months (**Figure** **4**), meaning that a higher ratio of looking at the eyes was associated with higher receptive vocabulary (note that a significant association between the EMI and receptive vocabulary was reported in Viktorsson et al. 2023, where we analyzed the whole sample without dividing it into monolinguals and bilinguals). There were no statistically significant differences between the beta estimates in the monolingual and bilingual group, for any of the associations.

**TABLE 6:** GEE analyses of the associations between EMI at 5 months and later language measures.

	Monolinguals			Bilinguals			Group comparisons *p* value
	β [95% CI]	*p* value	*N*	β [95% CI]	*p* value	*N*	
Receptive vocabulary (14 m)	0.15 [0.04; 0.27]	0.011^*^	344	0.02 [−0.33; 0.37]	0.910	66	0.493
Expressive vocabulary (24 m)	0.05 [−0.08; 0.19]	0.438	277	−0.09 [−0.60; 0.42]	0.724	56	0.603
Expressive vocabulary (36 m)	−0.01 [−0.13; 0.11]	0.867	298	−0.18 [−0.44; 0.07]	0.154	58	0.235

^*^p < 0.05.

As prompted by a reviewer, we also analyzed the associations between EMI and language measures at 24 and 36 months while controlling for earlier language abilities. Conclusions did not change (see **Table** **S4** for details).

As additional sensitivity analyses (that were not pre‐registered), we calculated the associations between EMI and later language measures again, including only infants with Swedish as the main language heard at home. The results were very similar (**Table** **7**).

**TABLE 7:** GEE analyses of the associations between EMI at 5 months and later language measures for children who heard Swedish as the main language at home.

	Monolinguals			Bilinguals		
	β [95% CI]	*p* value	*N*	β [95% CI]	*p* value	*N*
Receptive vocabulary (14 m)	0.16 [0.04; 0.28]	0.012^*^	322	0.07 [−0.45; 0.59]	0.777	50
Expressive vocabulary (24 m)	0.05 [−0.09; 0.19]	0.467	261	0.15 [−0.40; 0.70]	0.598	45
Expressive vocabulary (36 m)	−0.001 [−0.12; 0.11]	0.959	280	−0.16 [−0.45; 0.13]	0.279	44

^*^p < 0.05.

### 3.4 Additional Sensitivity Analyses

In three follow‐up analyses (not pre‐registered), we examined whether the degree of exposure to a second language at home, rather than the categorical classification of monolingualism versus bilingualism, was associated with each gaze measure. This approach allowed us to test whether bilingual language experience might affect gaze behavior in a continuous manner, which could be obscured when infants are grouped solely by language status. This was examined using GEE analyses, with sex and age regressed out of the measures before analysis. There were no significant associations between percentage of hearing a second language and EMI (*β* = −0.046, *p* = 0.630), ratio of looking at the face in the face pop‐out task (*β* = −0.044, *p* = 0.620), or latency of looking at the face in the face pop‐out task (*β* = 0.034, *p* = 0.601).

Because some infants were born prematurely (before week 37), we performed additional sensitivity analyses (not pre‐registered) where we estimated the mixed‐effects models for all gaze measures, including only infants who were born at week 37 or later. In total, these analyses included 318 monolinguals and 57 bilinguals. No difference between monolinguals and bilinguals were found for EMI (B = −0.088, SE = 0.054, t(181.90) = −1.643, *p* = 0.102, see **Table** **S5** for full results), ratio of looking at the face in the face pop‐out task (B = −0.001, SE = 0.023, t(182.22) = −0.055, *p* = 0.957, see **Table** **S6** for full results), or latency of looking at the face in the face pop‐out task (B = 0.002, SE = 0.262, t(185.59) = 0.009, *p* = 0.993, see **Table** **S7** for full results).

## 4 Discussion

The aim of this study was to examine whether, when viewing dynamic videos of an adult face, bilingual infants orient more to the mouth than monolingual infants, and whether this tendency at 5 months was differently related to language development in toddlerhood in the two groups. In addition, we examined whether bilingual and monolingual infants differed with regards to latency of first look to faces and ratio of looking at static faces interspersed with non‐social objects.

No statistically significant difference was found between monolinguals and bilinguals regarding the tendency to look at the eyes versus the mouth. However, descriptively, bilinguals looked more at the eyes than the monolinguals. This is not in line with the idea that bilingual infants rely on audiovisual speech cues more than monolingual infants, and therefore would attend earlier and to a higher degree to the mouth of a talking person (Pons et al. 2015). Although Pons et al. (2015) found that 21 bilingual infants attended more to the mouth of a talking face than 20 monolingual infants already at 4.5 months of age, Morin‐Lessard et al. (2019) did not find a significant difference between bilinguals and monolinguals at 5 months of age, in a study using a similar setup and sample size. It is notable that we did not find any differences between mono‐ and bilinguals in a sample of more than 400 monolingual and around 100 bilingual infants, but it is possible that the age of the infants included in the current study is not optimal for studying potential differences in attention to visual speech cues. As the attention to eyes generally declines between 4.5 and 6 months (Lewkowicz and Hansen‐Tift 2012), large individual variation at 5 months might reflect different levels of maturity in this gaze behavior (Viktorsson et al. 2023), which may obscure potential differences between monolinguals and bilinguals. It is also possible that bilingual infants do not attend to the mouth at an earlier age than monolinguals do, but rather keep focusing on the mouth at an older age than monolinguals do, reflecting a prolonged reliance on visual speech cues to help differentiate input from two languages. Thus, longitudinal studies following children beyond the first year of life will be important to determine whether bilingual children's attention to the mouth persists longer than that of monolingual children. Although some studies have focused on differences between monolingual and bilingual infants cross‐sectionally at different ages (e.g., Morin‐Lessard et al. 2019; Pons et al. 2015), future studies should focus on assessing differences longitudinally in the same sample.

Although we found a significant association between a tendency to look at the eyes at 5 months and receptive vocabulary at 14 months, this was only true for the monolingual children. The idea that bilingual infants attend more to the mouth than monolingual infants in order to get more visual speech cues would imply that there is an association between early mouth looking and later language in this group, but we found no association between the EMI and later language for bilingual children. It is worth noting that the beta estimates in the two groups were not significantly different from each other, and that the bilingual group was much smaller than the monolingual group (*n* = 66 and *n* = 344, respectively, for the association with receptive vocabulary at 14 months). This means that the power to find a significant association was higher in the monolingual group, and it is possible that a similar association would be found if we collected data on the same number of bilingual infants.

One important limitation for the interpretation of our findings is the use of online parent‐rated questionnaires to measure language development. The questionnaires listed Swedish words, and although the Words and Gestures form of CDI included an instruction to report words the child knows in any language, we cannot be certain that they followed this instruction throughout all of the questionnaires. It is worth noting, however, that very similar results were found when only including mono‐ and bilinguals who mainly heard Swedish at home. Nevertheless, future studies should include language measures more appropriate for bilingual children, or clarify the instructions to make it clear what the parents should report. Another focus of future research should be to measure the EMI in mono‐ and bilingual infants of slightly older ages, at the beginning of language acquisition, and potential differences in the association to later language.

Mercure et al. (2018) found a shorter latency of first look to faces and a larger number of fixations on faces in bilingual 4–10‐month‐olds, as compared to monolinguals. Here, we did not find any differences between mono‐ and bilinguals regarding latency of first look to faces or the mean ratio of looking at the face when interspersed with non‐social objects. Although Mercure et al. (2018) suggested that increased visual attention to still faces would allow bilinguals to take advantage of visual cues of articulation if the face was to begin producing speech, there is no clear theoretical rationale as to why this would lead to shorter latency of looking at static faces, or why the ratio of looking at faces would be higher in bilinguals. Faces attract attention from almost all infants (Portugal et al. 2024), and visual speech cues are found in the mouth area, not faces in general (and not in static faces). In addition, the tendency to look at faces is only weakly correlated with the tendency to look at eyes (vs. mouth), and while both behaviors are heritable, different genetic factors seem to be involved in the two (Falck‐Ytter 2024). It is worth noting that Mercure et al. (2018) found significant differences between monolinguals and bilinguals only when using a pooled sample from two different studies (and differently aged infants), but not when analyzing the samples separately.

A limitation of this study was the large variation in languages the infants were exposed to. It has been found that 15‐month‐old bilinguals who are exposed to two phonetically similar languages in their daily life attend more to the mouth than bilingual toddlers who are exposed to two distant languages in their daily life (Birules et al. 2019), suggesting that reliance on visual speech cues partly depends on the difficulty of the speech‐processing task. This might be true also at 5 months of age, which we were not able to control for in this sample. It is notable that Pons et al. (2015) found a significant difference between monolingual and bilingual 4‐month‐olds when including bilinguals who heard Spanish and Catalan at home (i.e., two phonetically similar languages), while Morin‐Lessard et al. (2019) did not find any difference at 5 months when studying bilinguals who heard English and French at home (i.e., two languages that are quite distant from each other). Due to the large variation in language pairs within our bilingual sample, we cannot draw definitive conclusions about the role of linguistic distance in our study. Future studies should aim to replicate earlier findings in samples of bilingual infants hearing either close or distant languages, comparing their gaze behavior to monolingual infants. It has also been argued that bilingualism should not be viewed as a categorical variable (e.g., Luk and Bialystok 2013), which may be particularly true in a sample as diverse as the one in the current study. It should be noted, however, that we found no association between the percentage of hearing a second language at home, regardless of the classification of monolingualism or bilingualism, and any of the gaze measures.

Another limitation is the common occurrence of preterm birth in twin pregnancies, and the potential effect on attention to dynamic faces. A recent study found that both very preterm infants (gestational age of 28 < 32 weeks) and moderate to late preterm infants (gestational age of 32 < 37 weeks) showed similar face scanning patterns when viewing a person speaking either their native or non‐native language at 8 months of age (Berdasco‐Muñoz et al. 2019). On the other hand, full‐term infants looked more at the mouth in the non‐native condition than in the native condition. In the current study, the mean gestational age was quite low (∼36.7 weeks). However, a sensitivity analysis including only full‐term infants (37 weeks or later) produced very similar results to analyses including the whole sample.

## 5 Conclusions

It has been hypothesized that bilingual infants, due to the challenge of learning two languages simultaneously, attend more to visual speech cues (i.e., a talking mouth) than monolingual infants. Here, in a sample of more than 400 monolingual and 100 bilingual infants, we did not find a difference between monolingual and bilingual 5‐month‐olds regarding the tendency to look at the eyes or mouth of dynamic faces. In addition, there was no statistically significant difference in the association between looking at eyes versus mouth and later language. Furthermore, no difference was found between the two groups regarding latency or ratio of looking at static faces interspersed with non‐social objects. In conclusion, we found no differences in attention to social stimuli in monolingual and bilingual infants, suggesting that both groups attend equally to communicative cues at 5 months of age.

## Author Contributions

The hypotheses and goals of this study were conceptualized by Charlotte Viktorsson and Terje Falck‐Ytter. Data were analyzed by Charlotte Viktorsson. Charlotte Viktorsson drafted the manuscript, and both authors reviewed, edited, and approved the final manuscript for submission.

## Funding

This research was funded by Riksbankens Jubileumsfond, Stiftelsen Sunnerdahls Handikappfond, and the Knut and Alice Wallenberg Foundation. Any views expressed are those of the author(s) and not necessarily those of the funders. The funders had no role in the design of the study; in the collection, analyses, or interpretation of data, in the writing of the manuscript, or in the decision to publish the results.

## Ethical statement

The authors assert that all procedures contributing to this work comply with the ethical standards of the relevant national and institutional committees on human experimentation and with the Helsinki Declaration of 1975, as revised in 2008. Parents gave informed consent to take part, and the study was approved by the regional ethics board in Stockholm.

## Conflicts of Interest

The authors declare no competing interests.

## Supporting information




**Supporting File 1**: desc70117‐sup‐0001‐SupMat.docx

## Data Availability

The analyses presented here were preregistered (https://osf.io/bnyqe/). The data and code necessary to reproduce the analyses presented here are not publicly accessible, but will be made available upon reasonable request to the corresponding author. Note that sharing of pseudonymized personal data will require a data sharing agreement, according to Swedish and EU law.
