# Supplementary material for: No Difference in Face Scanning Patterns Between Monolingual and Bilingual Infants at 5 Months of Age
Source: Dev Sci. 2025 Dec 30;29(2):e70117. doi: 10.1111/desc.70117 (PMC12750410; doi:10.1111/desc.70117)
Supplement: Supplementary file 1 — Supporting File 1: desc70117‐sup‐0001‐SupMat.docx [file DESC-29-e70117-s001.docx]

Supplementary Information

**No difference in face scanning patterns between monolingual and bilingual infants at five months of age**

Charlotte Viktorsson^1^ & Terje Falck-Ytter^1,2^

^1^Development and Neurodiversity Lab, Department of Psychology, Uppsala University; Uppsala, Sweden

^2^Center of Neurodevelopmental Disorders (KIND), Division of Neuropsychiatry, Department of Women’s and Children’s Health, Karolinska Institutet, Stockholm, Sweden

*Corresponding author. Email: charlotte.viktorsson@psyk.uu.se


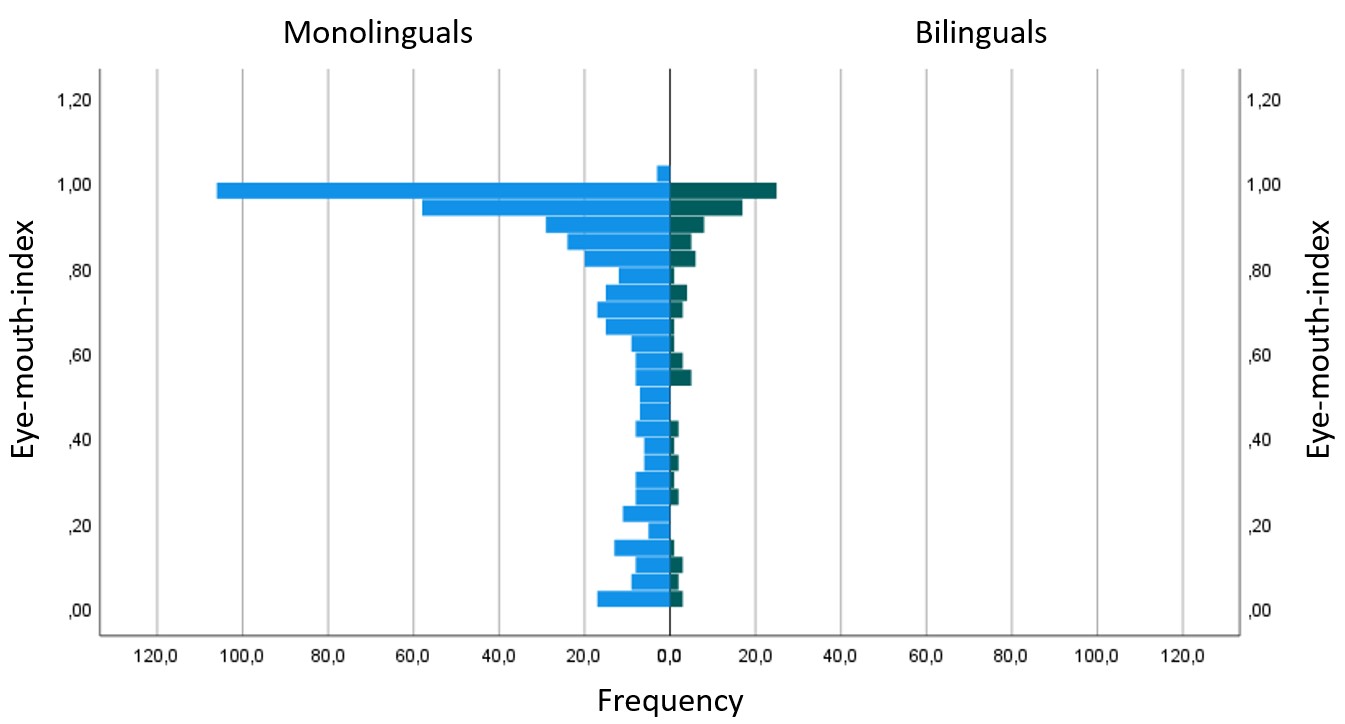


**Figure S1**. Distributional plot of the eye-mouth-index (EMI) in monolinguals (n = 437) and bilinguals (n = 96).


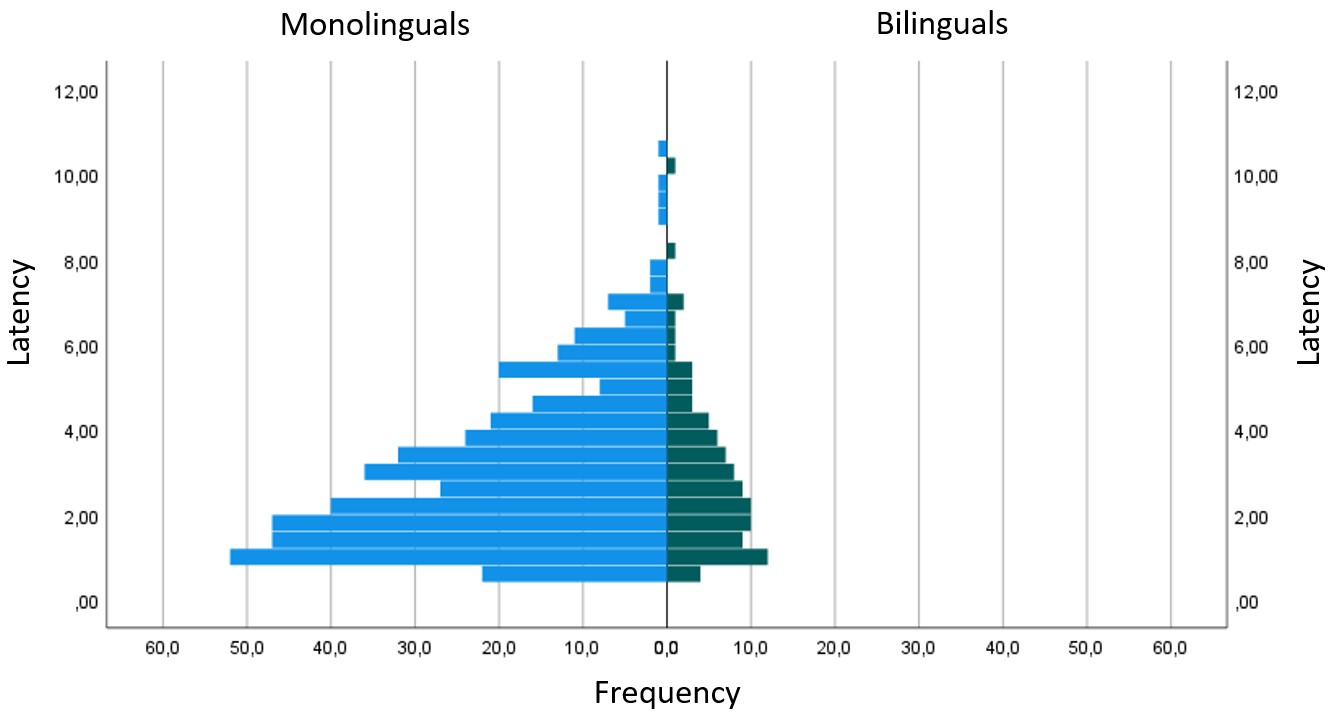


**Figure S2**. Distributional plot of latency of first look to faces in the face pop-out task, for monolinguals (n = 436) and bilinguals (n = 96).


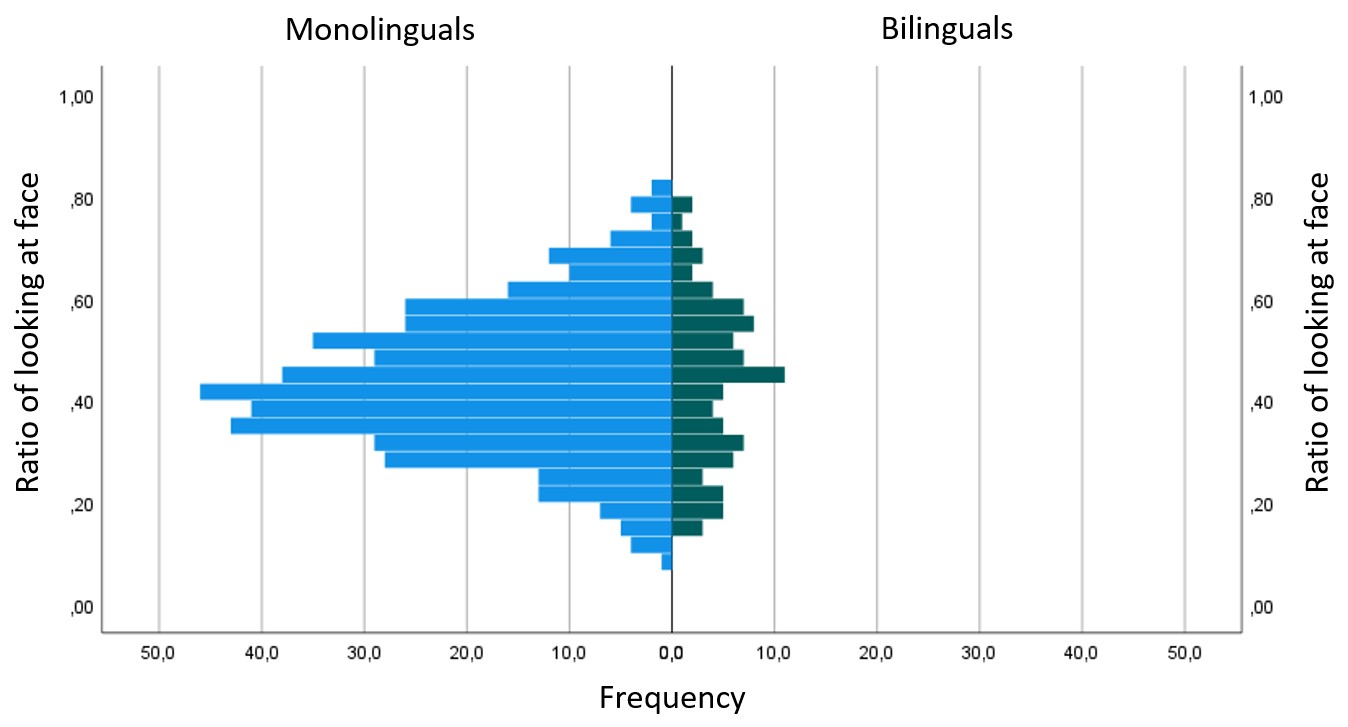


**Figure S3**. Distributional plot of ratio of looking at the face in the face pop-out task, for monolinguals (n = 436) and bilinguals (n = 96).

**Table S1**. Results of the linear mixed effects model predicting EMI, including only infants who heard Swedish as the main language at home.

| Predictor | Estimate | SE | df | t | p-value | 95% CI |
| --- | --- | --- | --- | --- | --- | --- |
| Intercept | 0.353 | 0.337 | 245.201 | 1.046 | 0.297 | -0.312; 1.017 |
| Language status | -0.059 | 0.048 | 238.583 | -1.226 | 0.221 | -0.154; 0.036 |
| Sex | 0.024 | 0.033 | 245.127 | 0.718 | 0.473 | -0.042; 0.090 |
| Age | 0.002 | 0.002 | 244.604 | 1.150 | 0.251 | -0.002; 0.006 |

Note. Model fit: -2LL = 200.59

**Table S2**. Results of the linear mixed effects model predicting ratio of looking at the face in the face pop-out task, including only infants who heard Swedish as the main language at home.

| Predictor | Estimate | SE | df | t | p-value | 95% CI |
| --- | --- | --- | --- | --- | --- | --- |
| Intercept | 0.128 | 0.153 | 243.243 | 0.840 | 0.402 | -0.172; 0.429 |
| Language status | 0.005 | 0.022 | 241.650 | 0.221 | 0.833 | -0.039; 0.048 |
| Sex | -0.019 | 0.015 | 241.387 | -1.270 | 0.205 | -0.049; 0.011 |
| Age | 0.002 | 0.001 | 243.741 | 2.249 | 0.025 | 0.000; 0.004 |

Note. Model fit: -2LL = -518.02

**Table S3**. Results of the linear mixed effects model predicting latency of looking at the face in the face pop-out task, including only infants who heard Swedish as the main language at home.

| Predictor | Estimate | SE | df | t | p-value | 95% CI |
| --- | --- | --- | --- | --- | --- | --- |
| Intercept | 7.802 | 1.828 | 223.998 | 4.267 | <.001 | 4.198; 11.405 |
| Language status | 0.054 | 0.264 | 224.157 | 0.206 | 0.837 | -0.467; 0.576 |
| Sex | 0.145 | 0.183 | 223.874 | 0.793 | 0.429 | -0.215; 0.504 |
| Age | -0.031 | 0.011 | 224.496 | -2.878 | 0.004 | -0.051; -0.010 |

Note. Model fit: -2LL = 1919.41

**Table S4**. GEE analyses of the associations between EMI at 5 months and language measures at 24 and 36 months, when controlling for earlier language abilities.

|  | **Monolinguals** | | | **Bilinguals** | | |
| --- | --- | --- | --- | --- | --- | --- |
|  | β [95% CI] | p-value | N | β [95% CI] | p-value | N |
| Expressive vocabulary (24m) | 0.04 [-0.09; 0.17] | 0.540 | 257 | -0.02 [-0.35; 0.30] | 0.891 | 48 |
| Expressive vocabulary (36m) | -0.09 [-0.19; 0.01] | 0.066 | 245 | -0.12 [-0.40; 0.15] | 0.390 | 47 |

**Table S5**. Results of the linear mixed effects model predicting EMI, including only infants who were born in week 37 or later.

| Predictor | Estimate | SE | df | t | p-value | 95% CI |
| --- | --- | --- | --- | --- | --- | --- |
| Intercept | 0.192 | 0.402 | 178.993 | 0.479 | 0.633 | -0.601; 0.986 |
| Language status | -0.088 | 0.054 | 181.898 | -1.643 | 0.102 | -0.195; 0.018 |
| Sex | 0.003 | 0.002 | 178.520 | 0.178 | 0.178 | -0.001; 0.008 |
| Age | 0.041 | 0.039 | 179.935 | 0.298 | 0.298 | -0.036; 0.117 |

Note. Model fit: -2LL = 145.47

**Table S6**. Results of the linear mixed effects model predicting ratio of looking at the face in the face pop-out task, including only infants who were born in week 37 or later.

| Predictor | Estimate | SE | df | t | p-value | 95% CI |
| --- | --- | --- | --- | --- | --- | --- |
| Intercept | 0.125 | 0.170 | 177.267 | 0.735 | 0.463 | -0.210; 0.459 |
| Language status | -0.001 | 0.023 | 182.216 | -0.055 | 0.957 | -0.047; 0.044 |
| Sex | -0.017 | 0.017 | 177.382 | -1.057 | 0.292 | -0.050; 0.015 |
| Age | 0.002 | 0.001 | 177.608 | 2.220 | 0.028 | 0.000; 0.004 |

Note. Model fit: -2LL = -422.54

**Table S7**. Results of the linear mixed effects model predicting latency of looking at the face in the face pop-out task, including only infants who were born in week 37 or later.

| Predictor | Estimate | SE | df | t | p-value | 95% CI |
| --- | --- | --- | --- | --- | --- | --- |
| Intercept | 6.965 | 1.915 | 178.872 | 3.637 | <.001 | 3.186; 10.745 |
| Language status | 0.002 | 0.262 | 185.588 | 0.009 | 0.993 | -0.514; 0.518 |
| Sex | -0.008 | 0.187 | 179.752 | -0.042 | 0.966 | -0.376; 0.360 |
| Age | -0.025 | 0.011 | 179.252 | -2.323 | 0.021 | -0.047; -0.004 |

Note. Model fit: -2LL = 1359.34
